# Characterization of the Complete Mitochondrial Genome of *Nibea chui*: Resolving a Taxonomic Controversy and New Phylogenetic Insights into Sciaenidae

**DOI:** 10.3390/biology15070544

**Published:** 2026-03-28

**Authors:** Chuanhao Chen, Ang Li, Shufang Liu

**Affiliations:** 1 College of Fisheries and Life Science, Dalian Ocean University, Dalian 116023, China; henchuanhao111@sina.com; 2 State Key Laboratory of Mariculture Biobreeding and Sustainable Goods, Yellow Sea Fisheries Research Institute, Chinese Academy of Fishery Sciences, Qingdao 266071, China; liang@ysfri.ac.cn

**Keywords:** *Nibea chui*, *Nibea coibor*, mitochondrial genome, Sciaenidae, phylogenetics, species delimitation, taxonomic relationship

## Abstract

*N. chui* is an economically important marine fish species distributed along the coastal waters of China, renowned for its delicate flesh texture and high-quality dried swim bladder. However, its scientific name and taxonomic relationship with *N. coibor* have long remained controversial, hindering accurate resource assessment and germplasm management. To address this issue, we sequenced and annotated the first complete mitochondrial genome of *N. chui* (GenBank accession: PZ024444). The circular mitogenome is 16,504 bp in length and contains 37 typical genes, with gene arrangement, nucleotide composition (A + T content: 52.07%), and codon usage patterns consistent with general teleost characteristics. Phylogenetic analyses based on 13 concatenated protein-coding genes revealed that *N. chui* and *N. coibor* form a maximally supported monophyletic clade (bootstrap support = 100%), with a pairwise genetic distance of 0. These mitochondrial results strongly suggest that the two nominal taxa are very closely related and may represent the same species. However, formal taxonomic synonymy cannot be established on mitochondrial evidence alone and requires further evaluation through examination of type material and comparative morphological study. Gene-specific selection pressure analyses showed that most mitochondrial protein-coding genes were subject to purifying selection, with ATP8 exhibiting the highest mean ω among genes with ω < 1, whereas *ND5* and *ND6* showed elevated ω values that warrant cautious interpretation. This study provides essential mitochondrial genomic resources for future research on species delimitation, phylogeny, and conservation of this important sciaenid fish.

## Simple Summary

The taxonomic status of *Nibea chui*, an economically important marine fish in China, has long been confused with *Nibea coibor*. In this study, we sequenced the complete mitochondrial genome of *N. chui* for the first time. Comparative mitochondrial analyses showed that *N. chui* and the published sequence of *N. coibor* are highly similar, and the concatenated sequences of the 13 mitochondrial protein-coding genes (PCGs) yielded a genetic distance of zero. However, because formal taxonomic synonymy requires integrative evidence, including examination of type specimens and comparative morphology, our results should be regarded as molecular support for a close taxonomic relationship rather than a definitive taxonomic revision. This study provides important genomic data for future systematic, conservation, and aquaculture research on this valuable sciaenid fish.

## 1. Introduction

*Nibea chui* (Osteichthyes: Perciformes: Sciaenidae) is a large demersal, warm-water fish widely distributed in the South China Sea, East China Sea, and Taiwan Strait. This species is renowned for its delicate flesh texture and delicious taste, while its dried swim bladder product (“white fish maw”) commands high nutritional and economic value, particularly in Guangdong and Hong Kong markets [1,2]. Consequently, *N*. *chui* has been recognized as a promising candidate for marine ranching and aquaculture development in southern China [3].

Despite its significant economic and ecological importance, the taxonomic status of *N. chui*—particularly its relationship with *Nibea coibor* Hamilton, 1822—has remained contentious for decades. Trewavas [4] first described *N*. *chui* in 1971, questioning the validity of earlier identifications of *N*. *coibor* in Chinese waters. Hamilton [5] originally described *N*. *coibor* from the Ganges River in 1822, but subsequent taxonomic revisions suggested that specimens identified as *N*. *coibor* in China might represent a distinct species [4,6]. This ambiguity has led to inconsistent usage of these two names in scientific literature, fisheries statistics, and aquaculture records [3,7,8,9,10], seriously impeding accurate resource assessment, germplasm management, and conservation planning.

The family Sciaenidae (drums and croakers) is among the most economically important marine fish families globally, yet it is characterized by high morphological similarity among congeners, complicating traditional taxonomic classification based on external characters [11]. Molecular approaches, particularly analysis of mitochondrial genomes, have emerged as powerful tools for resolving such taxonomic uncertainties. The mitochondrial genome offers several advantages for phylogenetic studies: maternal inheritance, lack of recombination, structural conservation, and a moderate evolutionary rate that provides sufficient resolution for species-level and deeper phylogenetic analyses [12,13,14]. To date, complete mitochondrial genomes have been reported for numerous sciaenid species, including *Otolithes ruber* [15], *Nibea albiflora* [16], and *Larimichthys crocea* [17]. However, a complete mitochondrial genome unequivocally attributed to the valid species *N. chui* remains unavailable. This gap is particularly critical given the pivotal phylogenetic position of the genus *Nibea* within Sciaenidae; without these data, robust resolution of intergeneric relationships at the family level and clarification of the evolutionary history of the genus remain unachievable.

Therefore, to clarify the phylogenetic position of *N. chui* within Sciaenidae and to re-evaluate its molecular relationship with *N. coibor*, this study aimed to: (1) sequence, assemble, and annotate the complete mitochondrial genome of *N. chui* for the first time, and comprehensively analyze its structural and molecular evolutionary characteristics; (2) reconstruct a phylogenetic framework of major Sciaenidae species distributed in Chinese coastal waters based on mitochondrial genomic data, thereby assessing the molecular affinity between *N. chui* and *N. coibor*; and (3) assess evolutionary selection pressures on its mitochondrial protein-coding genes, thereby revealing their evolutionary constraints and adaptive patterns. The results provide new mitochondrial evidence relevant to species delimitation and phylogenetic inference, and establish a foundation for future integrative taxonomic studies combining morphology, type-specimen examination, and nuclear genomic data.

## 2. Materials and Methods

### 2.1. Sample Collection and DNA Extraction

Specimens of *N. chui* were collected from the cultured population maintained by Huangjing Marine Biotechnology Co., Ltd. (Huizhou, China). The cultured stock was originally established from broodfish collected from Fujian coastal waters, which are within the known distribution range of *N. chui*. Approximately 0.5 g of pectoral muscle tissue was excised from each specimen, immediately preserved in 95% ethanol, and transported to the laboratory under −20 °C conditions. Samples were then stored at −80 °C until further use.

Total genomic DNA was extracted using a Marine Animal Tissue Genomic DNA Extraction Kit (Tiangen Biotech Co., Ltd., Beijing, China) following the manufacturer’s instructions. DNA quality and integrity were assessed using 1% agarose gel electrophoresis, and DNA concentration was measured with a Nano spectrophotometer.

### 2.2. Library Construction and DNA Sequencing

High-quality DNA samples were selected for library construction. Paired-end sequencing (150 bp) was performed on the DNBSEQ platform (MGI Tech Co., Ltd., Shenzhen, China). The raw sequencing data were subjected to quality control using SOAPnuke (v1.5.3) [18] to remove adapter sequences and low-quality reads, retaining high-quality clean reads for subsequent assembly.

### 2.3. Mitochondrial Genome Assembly, Annotation, and Analysis

The complete mitochondrial genome of *N. chui* was assembled de novo using NOVOPlasty (v4.3.5) [19], with the *COI* gene sequence obtained in this study used as the seed.

Genome annotation was conducted using the MITOS2 (http://mitos2.bioinf.uni-leipzig.de/index.py, accessed on 12 November 2025) [20] web server under the vertebrate mitochondrial genetic code. The circular mitochondrial genome map was visualized using MitoFish (http://mitofish.aori.u-tokyo.ac.jp/, accessed on 20 November 2025) [21].

Nucleotide composition and AT/GC skew values were calculated using MEGA X (v10.0.5) [22]. Codon usage bias and relative synonymous codon usage (RSCU) were analyzed using DNAMAN (v9) [23]. Secondary structures of tRNA genes were predicted using MITOS2 [20].

### 2.4. Phylogenetic Inference and Reconstruction

Complete mitochondrial genome sequences of 23 Sciaenidae species, together with two outgroup species (*Eleutheronema tetradactylum* and *Cephalopholis boenak*), were downloaded from the GenBank database (https://www.ncbi.nlm.nih.gov/genbank/, accessed on 27 November 2025) (Table 1). These two taxa were selected as outgroups because they are outside the family Sciaenidae and thus provide suitable external references for rooting the phylogenetic tree.

The nucleotide sequences of the 13 mitochondrial protein-coding genes (PCGs), with stop codons removed, were individually aligned and concatenated into a combined dataset.

MEGA X was used for pairwise genetic distance calculation based on the K2P model. IQ-Tree (v2.4.0) [24] was used to construct the ML tree with a codon position partition scheme, automatically selecting the best-fit evolutionary model (GTR + G + I), with parameters -bb 1000 and -alrt 1000 for 1000 ultrafast bootstrap replicates to assess branch support and SH-like aLRT test for branch reliability, respectively.

Pairwise genetic distances among species were calculated using the Kimura two-parameter (K2P) model based on the concatenated PCGs dataset.

### 2.5. Selection Pressure Analysis

To assess gene-specific selective pressure acting on the mitochondrial PCGs of *N. chui*, 11 related sciaenid species were selected for comparison: *Argyrosomus japonicus*, *Atrobucca nibe*, *Chrysochir aureus*, *Collichthys lucidus*, *Dendrophysa russelii*, *Johnius belangerii*, *Larimichthys polyactis*, *Miichthys miiuy*, *Otolithes ruber*, *Pennahia argentata*, and *Protonibea diacantha*. The 13 mitochondrial PCGs were analyzed separately. For each gene, sequences were aligned with the corresponding sequence of *N. chui*, and the nonsynonymous substitution rate (Ka), synonymous substitution rate (Ks), and their ratio (ω = Ka/Ks) were calculated using DnaSP v6.12.0 [25]. Mean ω values were then summarized for each gene across pairwise comparisons. Comparisons yielding undefined ω values, typically due to Ks = 0, were retained for inspection but excluded from mean ω summaries where appropriate. Selection pressure was interpreted as follows: ω < 1 indicates purifying selection, ω = 1 neutral evolution, and ω > 1 possible positive selection or relaxed purifying selection.

## 3. Results

### 3.1. General Features of the N. chui Mitogenome

**Figure 1:**
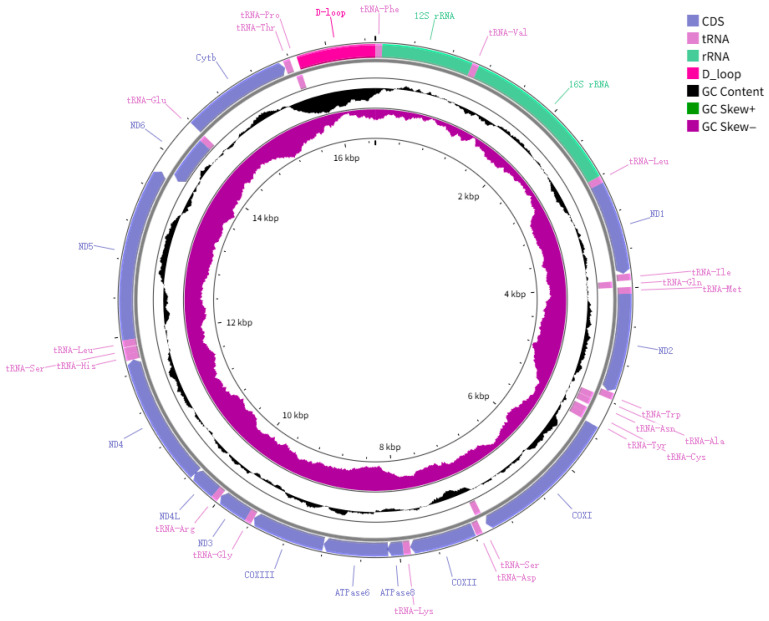
Circular map of the mitochondrial genome of *N. chui*. The outer and inner circles correspond to genes encoded by the heavy (H) strand and light (L) strand, respectively. Green indicates GC skew (+), while purple indicates GC skew (−).

The complete mitochondrial genome of *N. chui* was successfully sequenced and assembled. It is a typical closed-circular double-stranded DNA molecule with a total length of 16,504 bp (GenBank accession: PZ024444). The genome contains the standard 37 mitochondrial genes: 13 protein-coding genes (PCGs), 22 transfer RNA (tRNA) genes, 2 ribosomal RNA (rRNA) genes (12S rRNA and 16S rRNA), and one non-coding control region (D-loop). The gene order and transcriptional orientation are identical to those observed in most teleost fishes (Figure 1, Table 2).

Among the 37 genes, eight tRNA genes (*trnQ*, *trnA*, *trnN*, *trnC*, *trnY*, *trnS2*, *trnE*, and *trnP*) and the *ND6* gene are located on the light (L) strand, while all other genes are encoded on the heavy (H) strand. The total length of the 13 PCGs is 11,435 bp, accounting for 69.3% of the whole mitochondrial genome. The lengths of the *16S rRNA* and *12S rRNA* genes are 1705 bp and 953 bp, respectively, consistent with closely related species.

The overall nucleotide composition of the complete mitogenome is as follows: A = 26.89%, T = 25.18%, C = 31.63%, and G = 16.30%, with an A + T content (52.07%) slightly higher than the G + C content (47.93%). The genome exhibits a slight positive AT skew (0.01364) and a pronounced negative GC skew (−0.31984), a pattern commonly observed in other Sciaenidae species.

### 3.2. Characteristics of Protein-Coding Genes and Codon Usage

The 13 PCGs have a total A + T content of 50.91%. Among them, 11 genes use the conventional ATG as the initiation codon. Notably, the *ND1* and *ATP6* genes initiate with the alternative start codons GTG and CTG, respectively. For termination, the TAA codon is the most common. The *COI* gene terminates with AGA, while six genes (*ND2*, *COII*, *ATP6*, *COIII*, *ND3*, and *ND4*) employ incomplete stop codons (T or TA).

**Figure 2:**
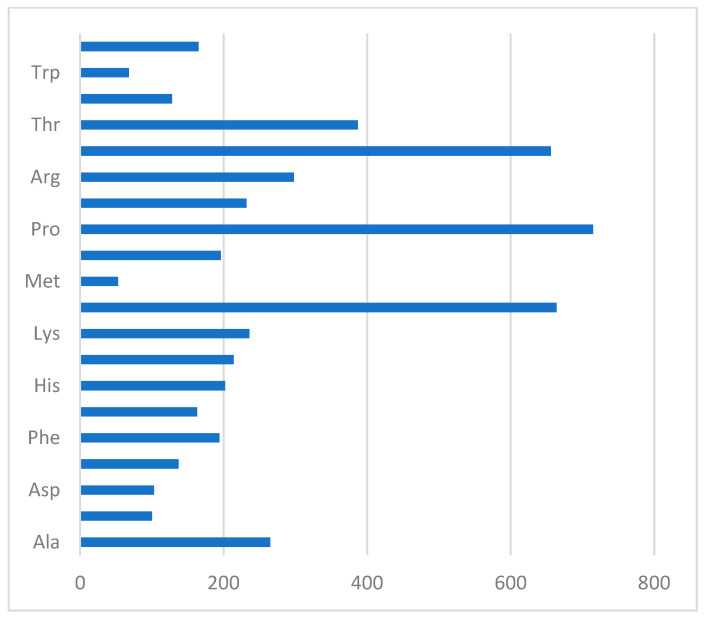
Amino acid usage in the mitochondrial genome of *N. chui*. The horizontal and vertical axes represent the number and types of amino acids, respectively.

**Figure 3:**
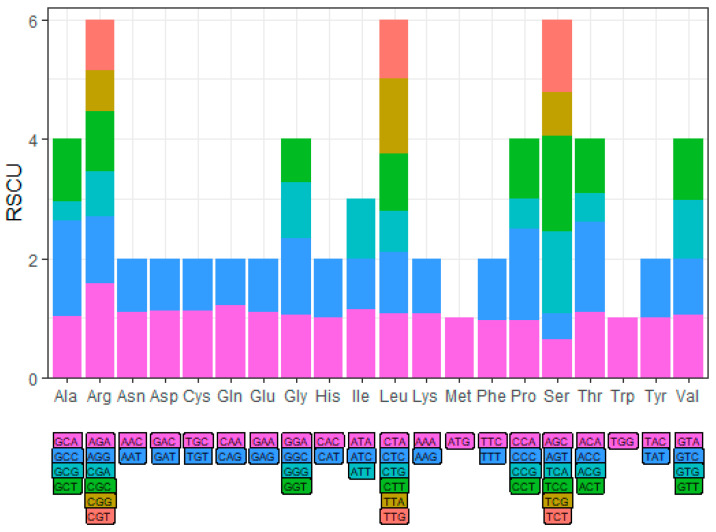
Relative Synonymous CodonUsage (RSCU) values of the mitochondrial genome of *N. chui*. Amino acids are shown on the horizontal axis, and the corresponding codons are displayed below.

These PCGs collectively encode 5176 amino acid residues. Proline (Pro), serine (Ser), leucine (Leu), and threonine (Thr) are the most abundant amino acids (Figure 2). Codon frequency analysis indicated that CCC (Pro), TCC (Ser), TTA (Leu), and ACC (Thr) were the most frequently used codons. RSCU analysis further revealed a distinct bias, with strong preferences for certain codons such as GCC (Ala) and AGA (Arg), while others like GCG (Ala) and CGG (Arg) were used rarely (Figure 3).

### 3.3. Selection Pressure (Ka/Ks) Analysis

Gene-specific Ka/Ks analyses of the 13 mitochondrial PCGs revealed that most genes in *N. chui* were subject to purifying selection when compared with 11 related sciaenid species (Table 3). Among genes with mean ω values below 1, *ATP8* showed the highest mean ω (0.1785), followed by *ND4* (0.1275) and *ND2* (0.0941), whereas *Cytb* (0.0244), *COIII* (0.0254), and *COI* (0.0308) exhibited the lowest values. In contrast, *ND5* and *ND6* yielded elevated mean ω values (2.2028 and 1.4383, respectively), indicating distinct evolutionary patterns relative to the other mitochondrial genes. In addition, several pairwise comparisons, particularly those involving *Chrysochir aureus*, produced undefined ω values because Ks was zero and were therefore interpreted cautiously.

### 3.4. Secondary Structure of tRNA Genes

**Figure 4:**
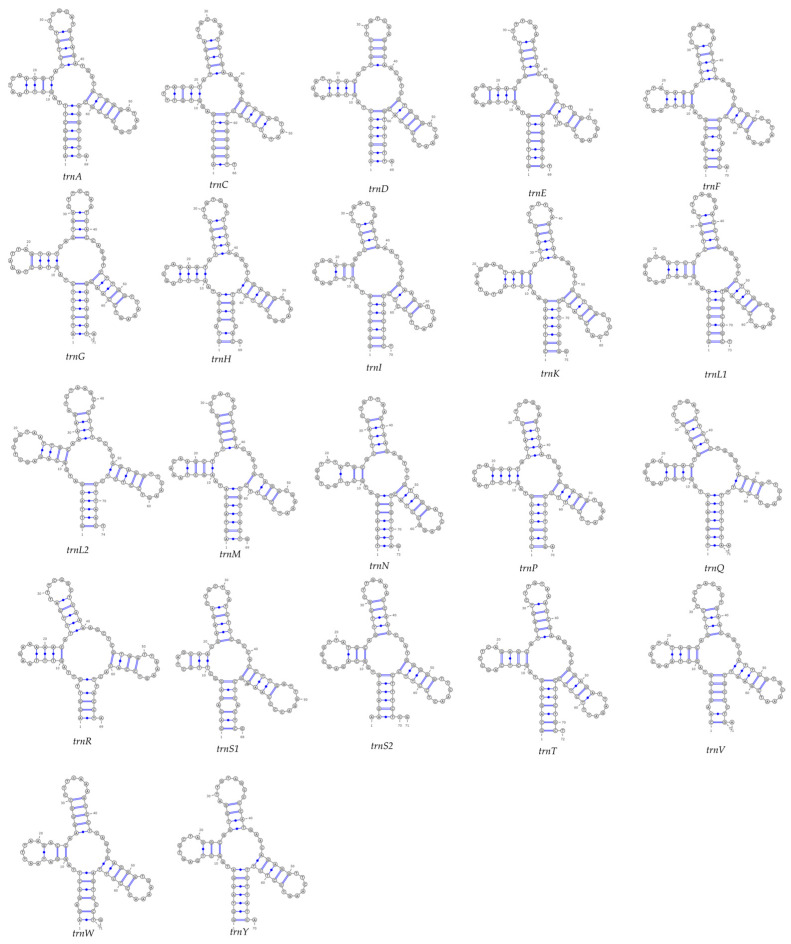
Predicted secondary structures of 22 tRNA genes in the mitochondrial genome of *N. chui*.

The 22 tRNA genes range in length from 66 to 73 bp. Secondary structure prediction confirmed that all 22 tRNAs can fold into the classic cloverleaf structures (Figure 4). Among them, 11 tRNAs (*trnA*, *trnC*, *trnE*, *trnG*, *trnK*, *trnL2* (TAA), *trnN*, *trnP*, *trnS2* (TGA), *trnW*, and *trnY*) non-Watson–Crick base pair (wobble pair) in their stems. These mismatches did not disrupt the overall stability of the secondary structures.

### 3.5. Phylogenetic Analysis and Species Delimitation

The genetic distance between the *N. chui* sequence obtained in this study and the GenBank sequence labeled as *N. coibor* (accession NC_025307.1) was calculated based on the PCGs (Table 4). The K2P genetic distance was 0 (indicating extremely close mitochondrial similarity between the two taxa).

**Figure 5:**
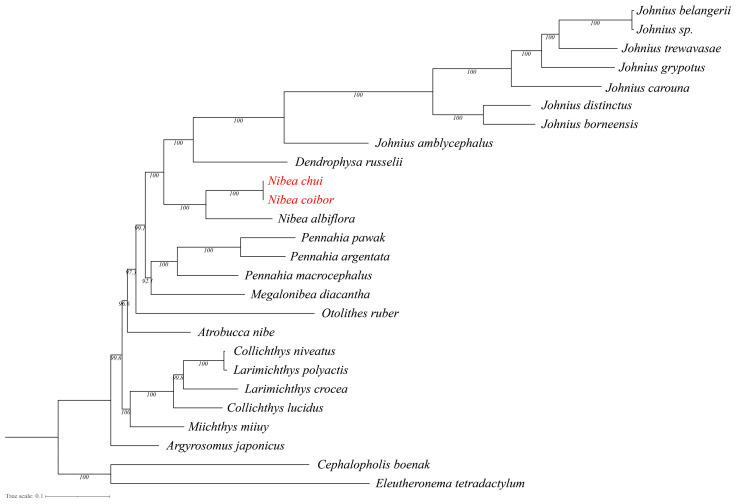
ML phylogenetic tree reconstructed from the concatenated sequences of 13 mitochondrial PCGs. Values at nodes indicate ultrafast bootstrap support. Note: The red color indicates the two fish species examined in this study.

The ML phylogenetic tree, reconstructed from the concatenated sequences of all 13 mitochondrial PCGs, yielded a highly resolved topology with strong nodal support (Figure 5). The tree delineates eight major clades within the Sciaenidae family. Notably, the sequences of *N. chui* (PZ024444) and *N. coibor* (NC_025307.1) formed a monophyletic clade with 100% bootstrap support, and the branch length within this clade was virtually zero. Together, these results indicate an extremely close mitochondrial relationship between the two nominal taxa and suggest that they may represent the same species. Nevertheless, this inference should be interpreted cautiously because mitochondrial data alone are insufficient for formal taxonomic synonymization.

## 4. Discussion

### 4.1. Structural and Evolutionary Characteristics of the N. chui Mitogenome

The mitochondrial genome of *N. chui* characterized in this study displays a high degree of structural conservation, typical of vertebrate mitogenomes. Its length (16,504 bp), standard complement of 37 genes, and gene arrangement are consistent with the general pattern observed in previously reported Sciaenidae species [13,26]. This structural uniformity further reinforces the reliability of mitochondrial genomes as stable and informative phylogenetic markers [27,28].

The observed nucleotide composition biases—a slight positive AT skew (+0.01364) and a pronounced negative GC skew (−0.31984)—are consistent with the asymmetric processes of heavy-strand replication and transcription characteristic of vertebrate mitochondria, reflecting underlying mutational and selective pressures [19,20]. The overall A + T content (52.07%) falls within the range documented for other sciaenids, indicating no significant compositional deviation [12,13].

Notable deviations from the standard vertebrate mitochondrial code were detected in the initiation codons of two PCGs. While the use of GTG in *ND1* is occasionally reported in fish mitogenomes, the employment of CTG as the start codon for *ATP6* is exceptionally rare [29,30]. This specific feature has been documented only sporadically in distantly related teleost lineages. Its presence in *N. chui* may reflect either a unique, fixed mutation within this lineage or a specialized mechanism such as RNA editing. Crucially, this rare trait was also identified in the *N*. *coibor* reference sequence (NC_025307.1), serving as a congruent, lineage-specific molecular signature that supports a close genetic relationship between the two specimens, independent of phylogenetic tree topology.

Codon usage analysis revealed a pronounced bias towards codons ending in C or G (e.g., GCC for Ala, AGA for Arg). This RSCU pattern reflects a non-random adaptation for translational efficiency and accuracy. Preferential utilization of codons corresponding to the most abundant tRNA species in the cellular pool enhances the speed and fidelity of protein synthesis—a selective pressure particularly intense for highly expressed genes encoding core cellular functions such as oxidative phosphorylation [23,24,25]. These findings underscore that even within a highly constrained genome, fine-scale sequence evolution is shaped by natural selection to optimize physiological performance.

### 4.2. Mitochondrial Evidence Supports a Close Taxonomic Relationship Between N. chui and N. coibor

The primary objective of this study was to clarify the ambiguous relationship between *N. chui* and *N. coibor* using newly generated mitochondrial genomic data. Our results consistently indicate an exceptionally close mitochondrial relationship between the *N. chui* mitogenome sequenced in this study and the published sequence labeled as *N. coibor* (NC_025307.1).

First, the pairwise K2P genetic distance based on the concatenated PCGs was 0, indicating no detectable divergence across the mitochondrial protein-coding dataset. Second, the two sequences formed a fully supported monophyletic clade with virtually zero branch length in the ML phylogeny. Third, both mitogenomes shared the same unusual genomic feature, namely the CTG start codon in *ATP6*, which provides additional lineage-specific molecular similarity. Taken together, these observations strongly suggest that *N. chui* and *N. coibor* are very closely related and may represent the same species.

Historically, the separation of *Nibea chui* and the taxon long recorded in Chinese literature as *N. coibor* was based mainly on traditional morphology-based taxonomy, in which closely related croakers were distinguished using external pigmentation patterns, relative fin positions, and other meristic or anatomical features. Under this framework, minor differences in body markings and fin proportions were treated as species-level characters, which likely contributed to the historical recognition of “Chu’s croaker” and “pale yellow croaker” as separate forms. However, later taxonomic reassessments indicated that much of this confusion was also nomenclatural rather than purely biological. Guo [9] noted that discrepancies among earlier Chinese classifications were largely attributable to numerous synonymies in coastal sciaenids, and specifically treated *Nibea chui* (Yuanding huangguyu) as a taxon whose name usage had been clarified through subsequent re-evaluation of genus- and species-level nomenclature. In addition, Guo listed “pale yellow croaker” as a historical synonym under *N. chui*, and also summarized earlier molecular work in which “yellow croaker” and “pale yellow croaker” clustered together. Taken together, this evidence suggests that the historical distinction between these two nominal taxa was likely influenced by reliance on subtle morphological characters and by the prolonged use of outdated names in the Chinese literature. Our mitochondrial results are therefore consistent with Guo’s reassessment in supporting a very close relationship between these taxa and the possibility that they represent the same species, although a definitive taxonomic decision still requires examination of type material and integrative evidence from morphology and nuclear markers.

However, we agree that mitochondrial evidence alone is not sufficient to support a formal taxonomic synonymization under a strict taxonomic framework. Because mitochondrial DNA reflects only the maternal lineage, definitive species-level taxonomic decisions should ideally be based on integrative evidence, including examination of name-bearing type material, detailed comparative morphology, and independent nuclear genomic markers. Therefore, the present study should be interpreted as providing strong molecular support for possible conspecificity between *N. chui* and *N. coibor*, rather than as a formal nomenclatural act.

Future work should focus on re-examining the type material of both nominal taxa, conducting detailed morphological comparisons, and incorporating genome-wide nuclear evidence, such as SNP-based analyses, to test species boundaries more rigorously. Such an integrative approach will be essential for determining whether the two names should ultimately be treated as synonyms within a formal taxonomic framework.

### 4.3. Gene-Specific Selection Patterns of Mitochondrial PCGs

Gene-specific Ka/Ks analyses showed that most mitochondrial PCGs of *N. chui* were under purifying selection, consistent with the strong functional constraints typically acting on vertebrate mitogenomes [26]. This overall pattern agrees with previous comparative mitogenomic studies, in which most mitochondrial genes exhibited ω values below 1, whereas a small subset of genes evolved relatively faster. In the present study, *ATP8* showed the highest mean ω among genes with ω < 1, while *COI*, *COIII*, and *Cytb* remained the most conserved. Similar patterns, with relatively elevated ω values in *ATP8* and low values in COX-family genes or *Cytb*, have been reported in other fish and metazoan mitochondrial genomes [27].

By contrast, *ND5* and *ND6* yielded mean ω values greater than 1 (2.2028 and 1.4383, respectively), indicating evolutionary patterns distinct from those of the other mitochondrial genes. These two genes encode subunits of NADH dehydrogenase (complex I) in the oxidative phosphorylation pathway and therefore play important roles in electron transport, proton translocation, and cellular energy metabolism [28]. From a functional perspective, elevated ω values in *ND5* and *ND6* may suggest that these genes have experienced either reduced purifying constraint or lineage-specific adaptive changes affecting mitochondrial energy conversion efficiency. In fishes, such changes could potentially be associated with environmental adaptation, because mitochondrial complex I performance is closely linked to aerobic metabolism and may influence physiological responses to environmental variation, particularly temperature and dissolved oxygen [29].

Nevertheless, these signals should be interpreted cautiously. Pairwise ω values greater than 1 do not, by themselves, provide definitive evidence of positive selection, because they may also reflect relaxed purifying selection, lineage-specific effects, or numerical instability in pairwise comparisons. This caution is particularly important for *ND6*, which is encoded on the light strand and is often reported to show distinct mutation and substitution patterns relative to other mitochondrial PCGs [30]. In addition, the relatively low mean Ks values of *ND5* and *ND6* may have amplified their ω estimates, and several pairwise comparisons, especially those involving *Chrysochir aureus*, produced undefined ω values because Ks = 0. Therefore, the present results support predominant purifying selection across the mitochondrial coding genome of *N. chui*, while suggesting that *ND5* and *ND6* may be candidate genes for further investigation of mitochondrial functional evolution and potential environmental adaptation. Future studies using codon-based site models, branch-site models, and broader population-level sampling will be necessary to determine whether these elevated ω values indeed reflect adaptive evolution.

### 4.4. Phylogenetic Insights and Implications for Sciaenidae Systematics

The phylogenetic tree based on complete mitochondrial genomes provides a solid basis for clarifying the evolutionary relationships among major lineages of Chinese coastal Sciaenidae. The well-supported clade formed by *Nibea*, *Otolithes*, and *Protonibea* (Clade V) indicates a shared evolutionary origin and offers a foundation for future morphological studies to identify potential common features of this group.

## 5. Conclusions

This study provides the first complete mitochondrial genome sequence for the economically important sciaenid fish, *N. chui*. The mitogenome is a typical circular molecule of 16,504 bp, encoding 37 genes with a structure and base composition consistent with related species.

Our mitochondrial analyses showed that *N. chui* and the published sequence labeled as *N. coibor* cluster together in a fully supported clade with negligible branch length and a genetic distance of zero based on the concatenated 13 mitochondrial protein-coding genes. These findings indicate an extremely close mitochondrial relationship and suggest that the two nominal taxa may represent the same species.

Nevertheless, because mitochondrial evidence alone is insufficient for formal taxonomic synonymization, the present study does not establish *N. coibor* as a junior synonym of *N. chui*. Instead, our results provide a molecular foundation for future integrative taxonomic research combining type-specimen examination, comparative morphology, and genome-wide nuclear markers such as SNPs. Overall, this work delivers an important genomic resource and contributes new evidence relevant to the systematics and conservation of Sciaenidae fishes.

## Figures and Tables

**Table 1:** Information of species used in the phylogenetic analysis.

No.	Species	Length (bp)	Accession Number	Note
1	*Nibea albiflora*	16,499	NC_015205.1	
2	*N. coibor*	16,502	NC_025307.1	
3	*Megalonibea diacantha*	16,521	NC_024573.1	
4	*Pennahia argentata*	16,486	KC545800.1	
5	*Pennahia macrocephalus*	16,508	NC_031409.1	
6	*Pennahia pawak*	16,408	NC_035942.1	
7	*Larimichthys crocea*	16,466	NC_011710.1	
8	*Larimichthys polyactis*	16,470	NC_013754.1	
9	*Collichthys lucidus*	16,442	NC_014350.1	
10	*Collichthys niveatus*	16,469	HM219223	
11	*Atrobucca nibe*	16,842	NC_035982.1	
12	*Johnius amblycephalus*	18,073	MF083698	
13	*Johnius belangerii*	19,154	NC_022464.1	
14	*Johnius borneensis*	18,630	NC_041308.1	
15	*Johnius carouna*	18,752	NC_035981.1	
16	*Johnius distinctus*	18,625	MF083699.1	
17	*Johnius grypotus*	18,523	NC_021130.1	
18	*Johnius trewavasae*	19,468	MF083700.1	
19	*Johnius sp.*	18,451	MG917694.1	
20	*Dendrophysa russelii*	16,626	NC_017606.1	
21	*Miichthys miiuy*	16,493	NC_014351.1	
22	*Argyrosomus japonicus*	16,496	NC_017610.1	
23	*Otolithes ruber*	16,589	NC_033909.1	
24	*Cephalopholis boenak*	16,771	KC537759.1	Outgroup
25	*Eleutheronema tetradactylum*	16,470	KC878730.1	Outgroup

**Table 2:** Structural features of the mitochondrial genome of *N. chui*.

Gene	Start	Stop	Size (bp)	Strand	Anticodon	Start Codon	Stop Codon	Continuity
*tRNA-Phe*	1	70	70	+	GAA			0
*12S rRNA*	71	1023	953	+				0
*tRNA-Val*	1024	1094	71	+	TAC			0
*16S rRNA*	1095	2799	1705	+				0
*tRNA-Leu2*	2800	2873	74	+	TAA			0
*ND1*	2874	3848	975	+		GTG	TAA	0
*tRNA-Ile*	3853	3922	70	+	GAT			4
*tRNA-Gln*	3922	3992	71	−	TTG			−1
*tRNA-Met*	3992	4060	69	+	CAT			−1
*ND2*	4061	5105	1045	+		ATG	T	0
*tRNA-Trp*	5106	5176	71	+	TCA			0
*tRNA-Ala*	5177	5245	69	−	TGC			0
*tRNA-Asn*	5248	5320	73	−	GTT			2
*tRNA-Cys*	5355	5420	66	−	GCA			34
*tRNA-Tyr*	5421	5490	70	−	GTA			0
*COXI*	5492	7048	1557	+		ATG	AGA	1
*tRNA-Ser2*	7044	7114	71	−	TGA			−5
*tRNA-Asp*	7118	7186	69	+	GTC			3
*COXII*	7195	7885	691	+		ATG	T	8
*tRNA-Lys*	7886	7960	75	+	TTT			0
*ATPase8*	7962	8129	168	+		ATG	TAA	1
*ATPase6*	8120	8802	683	+		CTG	TA	−10
*COXIII*	8803	9587	785	+		ATG	TA	0
*tRNA-Gly*	9588	9658	71	+	TCC			0
*ND3*	9659	10,007	349	+		ATG	T	0
*tRNA-Arg*	10,008	10,076	69	+	TCG			0
*ND4L*	10,077	10,373	297	+		ATG	TAA	0
*ND4*	10,367	11,747	1381	+		ATG	T	−7
*tRNA-His*	11,748	11,816	69	+	GTG			0
*tRNA-Ser*	11,817	11,884	68	+	GCT			0
*tRNA-Leu1*	11,890	11,962	73	+	TAG			5
*ND5*	11,963	13,801	1839	+		ATG	TAA	0
*ND6*	13,798	14,319	522	−		ATG	TAA	−4
*tRNA-Glu*	14,321	14,389	69	−	TTC			1
*Cytb*	14,389	15,531	1143	+		ATG	TAA	−1
*tRNA-Thr*	15,536	15,607	72	+	TGT			4
*tRNA-Pro*	15,613	15,682	70	−	TGG			5
D-loop	15,683	16,504	822	+				0

Note: “+” indicates the heavy strand; “–” indicates the light strand.

**Table 3:** Summary of gene-specific Ka, Ks, and ω (Ka/Ks) values for the 13 mitochondrial protein-coding genes (PCGs) of *N. chui*.

Gene	Mean Ka	Mean Ks	Mean ω
*ATP6*	0.0516	1.1804	0.0437
*ATP8*	0.1769	1.0019	0.1785
*COI*	0.0300	0.9577	0.0308
*COII*	0.0282	0.8559	0.0335
*COIII*	0.0192	0.7354	0.0254
*Cytb*	0.0216	0.8764	0.0244
*ND1*	0.0315	0.7614	0.0422
*ND2*	0.0785	0.8345	0.0941
*ND3*	0.0561	0.8436	0.0688
*ND4*	0.0596	0.8622	0.1275
*ND4L*	0.0388	0.7337	0.0550
*ND5*	0.3378	0.1579	2.2028
*ND6*	0.3040	0.2165	1.4383

**Table 4:** Genetic distance analysis based on the 13 PCGs.

	*N. chui*	*N. coibor*	*N. albiflora*	*P. pawak*	*P. macrocephalus*	*P. argentata*	*L. crocea*	*L. polyactis*	*A. nibe*	*A. japonicus*	*C. niveatus*	*C. lucidus*	*O. ruber*	*M. miiuy*	*M. diacantha*	*D. russelii*	*J. grypotus*	*J. carouna*	*J. distinctus*	*J. belangerii*	*J. borneensis*	*J. trewavasae*	*J.* sp.	*J. amblycephalus*
*N. chui*	0.00000	0.00000	0.15454	0.23978	0.20875	0.23229	0.23763	0.22832	0.20552	0.20454	0.22651	0.22500	0.26092	0.19977	0.21729	0.22343	0.38619	0.39774	0.35602	0.40110	0.36114	0.40120	0.39886	0.25798
*N. coibor*	0.00000	0.00000	0.15454	0.23978	0.20875	0.23229	0.23763	0.22832	0.20552	0.20454	0.22651	0.22500	0.26092	0.19977	0.21729	0.22343	0.38619	0.39774	0.35602	0.40110	0.36114	0.40120	0.39886	0.25798
*N. albiflora*	0.15454	0.15454	0.00000	0.24813	0.21336	0.24581	0.23934	0.23132	0.20593	0.20716	0.22846	0.22794	0.26446	0.20980	0.22489	0.22940	0.37891	0.39347	0.34432	0.39254	0.35495	0.39053	0.39272	0.26527
*P. pawak*	0.23978	0.23978	0.24813	0.00000	0.19898	0.12936	0.25109	0.25022	0.22167	0.22304	0.24835	0.24288	0.27477	0.22357	0.23037	0.25469	0.39086	0.39824	0.35916	0.40065	0.35869	0.39698	0.40045	0.28230
*P. macrocephalus*	0.20875	0.20875	0.21336	0.19898	0.00000	0.19106	0.22320	0.21812	0.18908	0.19049	0.21595	0.21046	0.25887	0.18488	0.19723	0.22364	0.39437	0.40836	0.35490	0.40374	0.35648	0.40464	0.40460	0.26315
*P. argentata*	0.23229	0.23229	0.24581	0.12936	0.19106	0.00000	0.25202	0.23924	0.21352	0.21972	0.23754	0.23600	0.26968	0.21756	0.22457	0.24782	0.39009	0.40811	0.36178	0.40683	0.36440	0.39915	0.40726	0.28231
*L. crocea*	0.23763	0.23763	0.23934	0.25109	0.22320	0.25202	0.00000	0.12622	0.18833	0.18116	0.12421	0.14038	0.26967	0.17714	0.22935	0.25257	0.39027	0.39891	0.36178	0.39951	0.36160	0.39681	0.39996	0.27930
*L. polyactis*	0.22832	0.22832	0.23132	0.25022	0.21812	0.23924	0.12622	0.00000	0.18382	0.18134	0.00582	0.12654	0.26339	0.17475	0.22756	0.24101	0.39863	0.40634	0.36788	0.40655	0.37704	0.40126	0.40726	0.27549
*A. nibe*	0.20552	0.20552	0.20593	0.22167	0.18908	0.21352	0.18833	0.18382	0.00000	0.14893	0.18224	0.18563	0.24427	0.15442	0.19732	0.22473	0.38321	0.39205	0.35652	0.38693	0.35637	0.38760	0.38794	0.26044
*A. japonicus*	0.20454	0.20454	0.20716	0.22304	0.19049	0.21972	0.18116	0.18134	0.14893	0.00000	0.17916	0.17660	0.24615	0.14618	0.19340	0.22566	0.37728	0.38312	0.35039	0.38892	0.36045	0.37930	0.38982	0.25890
*C. niveatus*	0.22651	0.22651	0.22846	0.24835	0.21595	0.23754	0.12421	0.00582	0.18224	0.17916	0.00000	0.12486	0.26255	0.17188	0.22433	0.23943	0.39764	0.40498	0.36560	0.40637	0.37523	0.40161	0.40672	0.27322
*C. lucidus*	0.22500	0.22500	0.22794	0.24288	0.21046	0.23600	0.14038	0.12654	0.18563	0.17660	0.12486	0.00000	0.25679	0.16721	0.21939	0.23742	0.38477	0.39086	0.35800	0.40136	0.36477	0.39509	0.39999	0.26642
*O. ruber*	0.26092	0.26092	0.26446	0.27477	0.25887	0.26968	0.26967	0.26339	0.24427	0.24615	0.26255	0.25679	0.00000	0.24329	0.24799	0.27385	0.40340	0.40533	0.37293	0.40586	0.38332	0.40152	0.40594	0.30357
*M. miiuy*	0.19977	0.19977	0.20980	0.22357	0.18488	0.21756	0.17714	0.17475	0.15442	0.14618	0.17188	0.16721	0.24329	0.00000	0.19452	0.21782	0.39846	0.40747	0.36539	0.40916	0.36903	0.40091	0.41064	0.25859
*M. diacantha*	0.21729	0.21729	0.22489	0.23037	0.19723	0.22457	0.22935	0.22756	0.19732	0.19340	0.22433	0.21939	0.24799	0.19452	0.00000	0.23085	0.39973	0.41517	0.36714	0.41135	0.36532	0.40814	0.41226	0.26032
*D. russelii*	0.22343	0.22343	0.22940	0.25469	0.22364	0.24782	0.25257	0.24101	0.22473	0.22566	0.23943	0.23742	0.27385	0.21782	0.23085	0.00000	0.38005	0.38754	0.34175	0.38328	0.34311	0.38463	0.38294	0.25388
*J. grypotus*	0.38619	0.38619	0.37891	0.39086	0.39437	0.39009	0.39027	0.39863	0.38321	0.37728	0.39764	0.38477	0.40340	0.39846	0.39973	0.38005	0.00000	0.21056	0.25355	0.18734	0.25236	0.17045	0.18662	0.33984
*J. carouna*	0.39774	0.39774	0.39347	0.39824	0.40836	0.40811	0.39891	0.40634	0.39205	0.38312	0.40498	0.39086	0.40533	0.40747	0.41517	0.38754	0.21056	0.00000	0.25547	0.20962	0.25369	0.20252	0.20936	0.34944
*J. distinctus*	0.35602	0.35602	0.34432	0.35916	0.35490	0.36178	0.36178	0.36788	0.35652	0.35039	0.36560	0.35800	0.37293	0.36539	0.36714	0.34175	0.25355	0.25547	0.00000	0.26222	0.12836	0.24769	0.26141	0.31023
*J. belangerii*	0.40110	0.40110	0.39254	0.40065	0.40374	0.40683	0.39951	0.40655	0.38693	0.38892	0.40637	0.40136	0.40586	0.40916	0.41135	0.38328	0.18734	0.20962	0.26222	0.00000	0.25708	0.16448	0.00537	0.35620
*J. borneensis*	0.36114	0.36114	0.35495	0.35869	0.35648	0.36440	0.36160	0.37704	0.35637	0.36045	0.37523	0.36477	0.38332	0.36903	0.36532	0.34311	0.25236	0.25369	0.12836	0.25708	0.00000	0.25115	0.25669	0.31110
*J. trewavasae*	0.40120	0.40120	0.39053	0.39698	0.40464	0.39915	0.39681	0.40126	0.38760	0.37930	0.40161	0.39509	0.40152	0.40091	0.40814	0.38463	0.17045	0.20252	0.24769	0.16448	0.25115	0.00000	0.16352	0.34835
*J. sp.*	0.39886	0.39886	0.39272	0.40045	0.40460	0.40726	0.39996	0.40726	0.38794	0.38982	0.40672	0.39999	0.40594	0.41064	0.41226	0.38294	0.18662	0.20936	0.26141	0.00537	0.25669	0.16352	0.00000	0.35656
*J. amblycephalus*	0.25798	0.25798	0.26527	0.28230	0.26315	0.28231	0.27930	0.27549	0.26044	0.25890	0.27322	0.26642	0.30357	0.25859	0.26032	0.25388	0.33984	0.34944	0.31023	0.35620	0.31110	0.34835	0.35656	0.00000

## Data Availability

The mitochondrial genome supporting this study has been deposited in GenBank (http://www.ncbi.nlm.nih.gov) under the accession number PZ024444.

## Abbreviations

The following abbreviations are used in this manuscript:

aLRT	Approximate likelihood ratio test
K2P	Kimura two-parameter model
ML	Maximum Likelihood
mtDNA	Mitochondrial DNA
RSCU	Relative synonymous codon usage
PCGs	Protein-coding genes
